# Iodide of Potassium and Carb, Potash in Rheumatism

**Published:** 1863-11

**Authors:** John R. Flock

**Affiliations:** Assistant Surgeon, California Vols.


### Selections.

On the Use of Iodide of Potassium and Carb. Potash, given alternately in Rheumatism.—J. T—, æt. 40 years, was attacked with Syphilitic Rheumatism, of a very severe character, which rendered him entirely helpless; he had been under treatment for three months previous to my seeing him, with apparently no benefit arising from the treatment he had been subjected to. His appetite had been very poor; very restless at night; urine highly colored and scanty. All the joints of the lower extremities painful, particularly the right hip. I applied a blister over the hip, and gave tinct. gent. ʒij., carb. sodæ gr. x., three times daily, and continued this treatment for five days, with great improvement of the appetite. I then commenced with carb. potash, gr. xx., iod. potassium, gr. v., three times daily;—continued this treatment for five or six days, with no improvement; pain quite as severe as in the commencement of the treatment, except when I applied the blister; then the pain was not quite so severe.

I thought I would see what virtue there was in the preparation of potash, when given alone, and ordered of carb. potash gr. xx., three times a day, and was most agreeably surprised, at the end of the third day, to see such an improvement; but, at the end of the fifth day, I found the case retrograding, so I stopped the carb. potash, and commenced with the iodide of potassium, gr. v., three times daily, and was pleased to see a gradual improvement, the same as in the first instance, when I gave the carb. potash alone; so I kept alternating the treatment until I had a perfect cure. It will be perceived that the quantity of the medicine was in no way altered, only they were given separately.

Another case I will give. C. A—, æt. 30 years, suffering from Rheumatism to such an extent that, at times, he was unable to use a knife and fork at table. In this case, I had, from the commencement of the disease, used such treatment as is generally prescribed for such cases, with little or no improvement; I then tried the same remedies and in the same manner as in the first mentioned case, and the result was very satisfactory.

I have tried this treatment in some seven or eight cases, and the result has been uniformly the same. I would like to have this mode of treatment thoroughly tested by the medical men on this coast, and to hear the result. I do not pretend to give or lay down any theory why these medicines should give such satisfactory results when given alone, and have little or no effect when given conjointly, but such is the result in the cases I have treated.

JOHN R. FLOCK, M. D.,
Assistant Surgeon, California Vols.

---

THE following interesting item we translate from *Canstatt's Jahresbericht*,—last number issued,—which shows that, in the intercommunication between mother and offspring, even foreign matter may pass from the mother to the young, through milk as well as placental blood.

The fact having been previously noticed by Flourens, that the bones of the fœtus became colored red, when the mother has been fed upon coloring matter, he extended his observations still further, and has found that the bones of the young offspring become red-tinted when, during the period of nursing, its mother feeds upon reddened food. The experiment succeeded perfectly in young suckling pigs, of which the bones became red in from 14 to 20 days. Since, however, the pigs might have eaten some of the reddened food of the mother, Flourens selected another class of animals for experiment, in which this source of error could not exist, viz., albino rats and rabbits. In the albino rat, the skeleton became red in 11 days; in the albino rabbit, the same phenomenon occurred in 9 days; though not a trace of reddened matter had been eaten by the young, since they had lived wholly upon the milk of their mothers. (The coloring material usually employed in these experiments is that from the Rubia tinctoria, or madder.—*Editor San Francisco Med. Press.*)

